# Head-driven gender assignment in noun-noun compound recognition: Evidence from a determiner priming task in German

**DOI:** 10.1371/journal.pone.0348377

**Published:** 2026-05-20

**Authors:** Antje Lorenz, Elisabeth Beyersmann

**Affiliations:** 1 Department of Linguistics, Faculty of Linguistics and Literary Studies, Bielefeld University, Germany; 2 School of Psychological Sciences, Faculty of Medicine, Health and Human Sciences, Macquarie University, Sydney, Australia; City University of Hong Kong, HONG KONG

## Abstract

A visual lexical decision task with noun-noun compound targets (e.g., *Wasserflasche* [waterbottle]) and gender-marked determiner primes (der, die, das [the] for masculine, feminine, neuter]) was used to examine how compound words are stored at a lexical-syntactic level. It was tested whether the embedded constituents activate their grammatical gender separately. Determiner primes were either congruent with the compound’s morphological head (*die* - *Wasserflasche*), the compound’s modifier (*das* - *Wasserflasche*), or incongruent with both constituents (*der* – *Wasserflasche*). Data from two online experiments are reported. The experimental design was identical but prime durations slightly varied. Head-congruent determiner primes speeded compound recognition in both experiments, but no effects were observed for modifier-congruent primes. The results of both experiments suggest activation of the grammatical gender of the head constituent, whereas the modifier’s gender seems not to be activated. Evidence in line with constituent-specific processes of compounds was only observed in the analysis of response accuracies of Experiment 2, as indicated by significant interactions between semantic transparency and constituent-specific determiner-priming effects. Our data are in line with head-driven gender assignment during noun-noun compound recognition.

## Introduction

German is a morphologically rich language, and it is rife with morphologically complex words, such as compounds. German compounds are written as one word without spaces (e.g., *Mineralwasserflasche* [mineral water bottle]). Linguistically, compound words are built up from constituent morphemes that are the smallest units carrying meaning [1]. In psycholinguistic research, it is a matter of continuous debate whether access to these building blocks is always needed when compounds are processed in tasks, such as visual or auditory lexical decision or picture naming [2,3].

Empirical evidence in favor of morpheme-based lexical representations and processes is rich, both from studies that tested compound processing in comprehension [e.g., 4–13], as well as from studies testing compound production [e.g., 14–18, for contrasting data, see 19,20]. Notably, these studies mainly focus on compound words in isolation and on their representation at the word-form level of the mental lexicon. In contrast, type of representation and processing of compounds at a lexical-syntactic level (also called lemma) has been tested less frequently and the available empirical data are mixed. While some studies have reported evidence for the separate processing of a compound’s constituents at a lexical-syntactic level [21–25], other studies obtained no such evidence [e.g., 16,26,27].

The present study asked how the lexical-syntactic features of compound nouns are processed during visual word recognition. A primed lexical decision task with German noun-noun compound targets and gender-marked determiner primes was used to investigate the representation of compound words at a lexical-syntactic level of the orthographic lexicon. Materials and the experimental design were adapted from a previous picture-naming study [26].

The lexical decision task is a prominent tool in psycholinguistics to investigate lexical processes and representations during word recognition [28,29]. Word- and nonword stimuli are presented one by one in randomized order and participants are instructed to decide as quickly as possible whether a given letter string (or spoken stimulus) is a word of a given language. Lexical decision tasks are frequently combined with priming to investigate different levels of processes. In the current study, gender-marked determiner primes and noun-noun compound targets were used to tap into the representation of compounds at a lexical-syntactic level (also commonly referred to as ‘lemma level’).

German is a perfect language to investigate this research question because it has many compound nouns that carry grammatical gender (masculine, feminine, neuter). While there are some phonological and semantic regularities concerning the grammatical gender of German nouns, gender is not fully predictable from these features. Therefore, gender is assumed to be stored as a lexical-syntactic feature of nouns [30–32]. Because gender is overtly marked on the definite determiners of German nouns (*der, die, das* [the]), definite determiners can be used as lexical-syntactic primes to investigate noun processing [e.g., 33–35]. In German, grammatical features of morphologically complex words, such as noun-noun compounds, are determined by the morphological head, which is the rightmost constituent (e.g., *Blatt*_*neut*_ [leaf] in *Kleeblatt*_*neut*_ [cloverleaf] [36]). In contrast, the gender of the first constituent, the so-called modifier (e.g., *Klee*_*masc*_ in *Kleeblatt*) is irrelevant for the gender specification of the whole compound. Following this, for noun-noun compounds that differ in grammatical gender, such as *Klee*_*masc*_*blatt*_*neut*_*,* the modifier’s gender (e.g., masculine for *Klee*) should not modulate priming effects if the compound is accessed holistically (*Kleeblatt*_*neut*_*)* but modifier-effects would be expected if the compound’s constituents are accessed decompositionally (*Klee*_*masc*_*blatt*_*neut*_). Our study asked whether grammatical gender features of the underlying morphemes are activated at the level of the orthographic lexicon during visual compound recognition. A primed visual lexical decision task was used and three determiner priming conditions were implemented: 1) head-congruent prime (*e.g., das*_*neut*_ for *Klee*_*masc*_*blatt*_*neut*_), 2) modifier-congruent prime (*der*_*masc*_ for *Klee*_*masc*_*blatt*_*neut*_), and 3) incongruent prime (*die*_*fem*_ for *Klee*_*masc*_*blatt*_*neut*_).

### On the lexical representation of compound words: Theoretical and empirical considerations

The current study built on the assumption that during the visual recognition of morphologically complex words, both whole words and morphemes are accessed at the level of the orthographic lexicon, as predicted by Grainger and Beyersmann’s Word and Affix model [37,38]. Thus, a given compound will activate not just its lexical whole word representation (e.g., *farmhouse*), but also the lexical representations of its embedded morphemes (e.g., *farm* and *house*). This model is mainly based on evidence from masked primed lexical decision, testing the lexical representation of complex words at the word-form level. For example, it has been shown that under masked morphological priming conditions, significant facilitation in visual word recognition is observed with compound primes, overlapping in one constituent with the target word (e.g., *farmhouse-FARM*) relative to an orthographic control condition (e.g., *sandwich-SAND*), suggesting that readers rapidly decompose compound primes into their morphemic constituents [e.g., 5,9]. Interestingly, the priming effect is similar in size when semantically opaque compound primes are presented (e.g., *honeymoon-HONEY*), showing that compounds are decomposed, independently of semantics [e.g., 5,9]. Moreover, the recognition of compound word targets is facilitated by the prior presentation of a first-constituent prime (e.g., *milk-MILKMAN*) as much as by a second-constituent prime (e.g., *man-MILKMAN*), suggesting that both constituents of the compound word are activated during visual word recognition [e.g., 7]. Mechanisms of compound word segmentation are further supported by the results of [6], who manipulated the frequency of compounds and of their constituents in a visual lexical decision task. Robust constituent-frequency effects were obtained, that is participants were faster in the case of compounds with high vs. low frequency constituents, indicating decompositional processes during compound recognition [see also 39]). The authors also investigated compound nonwords (for related evidence, see [40]) and showed that effects were modulated by the type of nonword stimuli used in the lexical decision task. When two free morphemes were combined into a non-existing, but morphologically legal, novel compound, participants needed more time to reject this stimulus in the lexical decision task compared to legal pseudowords. Furthermore, when semantically opaque compound targets were included and presented with difficult non-words (novel compounds), reaction time was additionally delayed. Notably, however, the results point to decomposition of compound words prior to lexical access in both non-word conditions. Additional processes were likely used to classify these stimuli as non-words. The data were also modulated by the semantic transparency of existing compound words. While constituent frequency effects were obtained with opaque compounds, additional processing costs occurred due to the re-combination of separate morphemes for lemma or meaning access. These findings support the assumption that readers are sensitive to the morpho-orthographic structure of semantically transparent and opaque compound words [28,37,38,41–44].

Notably, the Word and Affix model does not differentiate between orthographic word-forms and lemmas [37,38]. To account for this, we drew on the predictions of the Two-Stage model, initially proposed for language production [45; see also 30,46], and later adapted for comprehension [e.g., 47,48]. Two separate levels as part of the mental lexicon are proposed here: a word-form and a lemma level. During visual word recognition the orthographic (input) word-form level and the lemma level are separate representations closely linked to each other [47–51]. The orthographic word-form level includes the corresponding morpho-orthographic properties of the written word, whereas the lemma level includes abstract lexical entries linked to lexical-syntactic properties, such as gender. Thus, in order to access grammatical gender of a noun, the lemma level needs to be accessed. The lemma level is viewed as modality-independent; that is, the same lemma level is accessed in comprehension of written and spoken words and in spoken production or spelling [e.g., 46,50,51]. Following this, the representation of compounds at lemma level may be investigated by means of comprehension or production tasks. Nevertheless, effects may differ between production and comprehension.

Two competing accounts exist that offer different explanations for the representation of compounds at a lexical-syntactic level: the single-lemma and multiple-lemma models. According to the single-lemma model, compounds are stored as a single (holistic) unit at the lemma level [e.g., 46], whereas according to the multiple-lemma model, a compound’s lemma consists of a complex entry, including the compound, that is directly linked to its’ underlying constituents at lemma level [25]. Notably, both accounts assume morpheme-sized units at the word-form level. Furthermore, while lemma representations exist for a compound’s constituents, as they are words, the critical difference between the two accounts is whether the constituent lemmas are part of the lexical representation of the compound’s lemma. This would imply that the embedded constituent-lemmas need to be retrieved in addition to a compound’s lemma during compound recognition or production, as predicted by the multiple-lemma representation account (for a more detailed description, see [25]).

Early evidence in favor of a multiple-lemma representation of compounds was provided by studies that tested the reading and naming of verb-noun (VN-) and noun-noun (NN-) compounds in participants with aphasia [13,23,25,52,53]. Marelli and colleagues [25] reported data from an Italian single-case study of an individual with aphasia and acquired dyslexia who suffered from a syntactic word-category deficit for verbs. This deficit was also present when VN-compound targets were presented, that are nouns overall. The participant showed a specific difficulty to retrieve the verbal constituent of VN-compounds during reading aloud and picture naming (e.g., lavapiatti [lit. wash dishes, dishwasher]). The data, thus, indicate separate retrieval of modifier and head at the lemma level where syntactic word category is coded in addition to other syntactic features, such as grammatical gender [54,55]. While this challenges the single-lemma model which predicts no syntactic effects of non-head constituents during compound reading or naming, these findings are consistent with the multiple-lemma model.

Mixed results have been obtained in picture-naming studies [e.g., 16,17,21,24,26]. Lorenz and colleagues [26] tested young neurotypical adults and presented gender-marked determiner primes in a picture naming task with noun-noun compound targets. They reported head-priming effects, but no modifier-priming effects in noun-noun compound production (picture-naming latencies). That is, participants were faster with head-congruent compared to incongruent determiner primes, whereas picture-naming latencies with modifier-congruent primes did not differ from incongruent primes. In contrast, in a subsequent study with the identical materials and design, older neurotypical speakers and participants with aphasia were tested and significant modifier-related priming effects were obtained, in line with a multiple-lemma representation of compounds [24]. Two different effects of modifier-congruency were observed when compared to an incongruent determiner-condition: 1) facilitatory effects, that is, faster compound production with modifier-congruent vs. -incongruent determiners were observed in older neurotypical speakers and in participants with aphasia who suffered from deficits of lexical access; 2) inhibitory effects, that is, longer picture naming latencies with modifier-congruent than -incongruent determiners were observed in participants with aphasia who suffered from post-lexical phonological deficits [24, Exp. 1]. It is still unresolved why modifier-priming effects have been observed in older and language-impaired speakers but not in young speakers. Presumably, type of processing was influenced by the automaticity and speed of compound activation and selection. Because the multiple-lemma model is a hybrid model, both holistic and constituent-specific access procedures may be used [for further discussion, see 24–26].

Empirical evidence from young adult speakers in favor of a multiple-lemma representation of compounds was provided by Döring et al. [21] who used a continuous picture-naming task with noun-noun compound targets. In this paradigm, participants name a seemingly random sequence of pictures, in which certain category members (e.g., mammals: *zebra, pony, cat, fox*) are presented for spoken picture naming. Cumulative semantic interference is observed in that naming latencies linearly increase with each new item of a category, an effect that is often related to lexical competition at the lemma level [e.g., 56,57]. The critical condition included subsets of seemingly unrelated noun-noun compounds with categorically related modifiers (e.g., modifier-condition, mammals: *zebra crossing, pony tail, cat litter, fox hole, …*). Cumulative semantic interference was observed for modifier-related compounds, pointing to the activation and retrieval of the modifier-constituent at the lemma level during compound production. Furthermore, the effect was modulated by the semantic transparency of compound targets. In particular, stronger interference was observed for compounds with transparent heads (e.g., *Fisch* in *Goldfisch*, fish in goldfish), whereas weaker interference was observed for compounds with more opaque heads (e.g., *Stich* in *Bienenstich* (literal: sting in “bee sting” (type of cake)), suggesting different representations and processes for semantically transparent vs. opaque compound words [21]. To sum up, prior evidence from different paradigms has revealed some inconsistencies concerning the processing and representation of compound words at lemma level, which has been shown to be influenced by both participant-related (age, language proficiency) and material-specific variables (e.g., semantic transparency). Moreover, while the representation of compound words at the lemma level has been mainly tested in production paradigms, it has only rarely been investigated in receptive tasks, such as a determiner-primed visual lexical decision task with compound targets (for auditory compound comprehension, see [22]).

### Gender-priming effects in word recognition: Empirical evidence

Previous studies have tested the effects of gender-marked determiner primes in different tasks and modalities with morphologically simple nouns, including auditory and visual lexical decision (e.g., [33,35,58–60]; for reviews, [49,61]). It has been found that word recognition with gender-congruent determiner primes is faster compared to word recognition with incongruent determiner primes. Interestingly, when a neutral control condition is included, congruent determiners sometimes fail to facilitate the response, whereas incongruent determiners usually delay the response (e.g., [35]; but see [58]; for a review, see [49]; for data from production, see [34,62]). These effects have been interpreted in terms of different levels of processing. While facilitatory priming effects have been attributed to gender priming at a lexical-syntactic level, inhibitory effects are typically interpreted as a result of a post-lexical checking mechanism (e.g., for comprehension, [33,35,58,63]; for production, see [34,64]). However, little is known regarding the role of gender processing in lexical decision and, to the best of our knowledge, there are no existing studies investigating gender constituent-priming effects in visual compound word recognition.

## The present study

The aim of the present study was to investigate how compound nouns and their grammatical gender are stored and processed at a lexical-syntactic level (lemma-level) during visual compound recognition [e.g., 48]. Two online visual lexical-decision experiments with 90 participants each were conducted. The materials and experimental design were adapted from Lorenz et al. [26] (Exp. 2). Each compound target was preceded by three different determiner primes: (1) determiner gender-congruent with the head (and with the compound as a whole), (2) determiner gender-congruent with the modifier, and (3) determiner neither gender-congruent with the head nor the modifier (see Table 1). The exact same experimental design was used across both Experiments 1 and 2. The only difference between the two experiments was that the prime duration slightly varied. Determiner primes were presented for 100 ms in Experiment 1, and for 200 ms in Experiment 2.

**Table 1:** Experimental conditions in Experiments 1 and 2, example.

Prime Condition	Prime	Target
**Head congruent**	das_neut_ [the]	Klee_masc_blatt_neut_ [cloverleaf]
**Modifier congruent**	der_masc_ [the]	
**Incongruent**	die_fem_ [the]	

In the case of a single-lemma representation of compounds, only the compound’s/ head’s gender is activated but not the gender of the compound’s modifier. Thus, head-priming effects (i.e., faster and more accurate responses with head-congruent vs. -incongruent primes) would be predicted but no modifier-related effects should be observed. In contrast, in the case of a multiple-lemma representation of compounds, constituent lemmas and the corresponding gender features are activated and priming effects would be expected for both head-congruent and modifier-congruent primes. However, because modifier-congruent primes are incongruent to the compound’s head, modifier-related effects may either be facilitatory or inhibitory when compared to a completely incongruent determiner [see 24 for compound production]. Finally, we further reasoned that the absence of any determiner priming effects would suggest that participants do not access grammatical properties of compound nouns during the lexical decision task.

The hypotheses, methods, and data analyses plan for the two experiments were preregistered (Experiment 1: https://aspredicted.org/LPP_VZN; Experiment 2: https://aspredicted.org/PM2_ZDB).

All corresponding materials, data and analysis scripts have been made available via the OSF repository (https://osf.io/9mpn7/?view_only=959019ec6c644d2ca4976359daeced78).

## Experiment 1

### Method

#### Participants.

Ninety adult participants (37 females; age: *M* = 31 years, *SD* = 8.3, min = 19 years, max = 51 years) were recruited from within Germany using Prolific (www.prolific.com). All participants were monolingual native German speakers, residing in Germany, with no learning impairments or history of neurological impairment. All participants gave their written consent to participate and were paid at a rate of £11.00/hour for their time. Experiment 1 was conducted in October 2022. The study received ethical approval by Humboldt-Universität zu Berlin (2022−51).

#### Materials.

The 240 stimuli for this lexical decision task included 90 compound targets, including 15 compound words consisting of a masculine modifier and feminine head (e.g., *Kaffee*_masc_*maschine*_fem_ [coffee machine]), 15 compound words consisting of a masculine modifier and neuter head (e.g., *Arm*_masc_*band*_neut_ [wrist band]), 15 compound words consisting of a feminine modifier and masculine head (e.g., *Tannen*_fem_*baum*_masc_ [pine tree]), 15 compound words consisting of a feminine modifier and neuter head (e.g., *Spinnen*_fem_*netz*_neut_ [spider web]), 15 compound words consisting of a neuter modifier and feminine head (e.g., *Wasser*_neut_*flasche*_fem_ [water bottle]), and 15 compound words consisting of a neuter modifier and masculine head (e.g., *Glas*_neut_*teller*_masc_ [glass plate]). In addition, 30 compound filler targets were included that included 10 masculine-masculine compound words (e.g., *Winter*_masc_*mantel*_masc_ [winter coat]), 10 feminine-feminine compound words (e.g., *Mause*_fem_*falle*_fem_ [mouse trap]), and 10 neuter-neuter compound words (e.g., *Bilder*_neut_*buch*_neut_ [picture book]). The head and modifier constituents were matched on word frequency, orthographic neighbourhood (Coltheart’s N), orthographic Levenshtein distance (Levenshtein N) and number of letters (see Table 2). Modifier frequency was numerically larger than head frequency (Table 2) and a t-test revealed a near-to significant difference (*p* = .053). Therefore, although not pre-registered, head and modifier frequency were included as covariates in the main analyses. The frequency and neighbourhood measures were retrieved from dlex-DB [65]. In addition, the semantic transparency between the head and modifier constituents was calculated. Semantic transparency values were extracted from fastText [e.g., 66–68] using the German model and the R package LSAfun [69]. The values represented the semantic similarity between each constituent (e.g., *Zirkus, Zelt* [circus, tent]) and its corresponding compound word (e.g., *Zirkuszelt* [circus tent]). It has been shown that fastText is able to successfully model some aspects of novel complex word processing [e.g., 70,71]. FastText was used here instead of rating data. This allowed us to control both existing and novel compounds for constituent-based semantic transparency. The extracted semantic transparency values for head and modifier constituents were similar (see Table 2), but not statistically matched, and therefore further explored in a set of non-preregistered post-hoc analyses.

**Table 2:** Mean item characteristics for head constituents, modifier constituents and compound words (standard deviations in parentheses).

	Heads	Modifiers	Compounds
**Word Frequency**	34.4 (54.4)	54.0 (96.3)	0.8 (1.2)
**Orthographic Neighbours (Coltheart’s N)**	26.3 (18.9)	24.9 (17.1)	0.4 (0.7)
**Orthographic Levenshtein Distance** **(Levenshtein N)**	36.6 (23.3)	35.6 (21.3)	1.7 (1.3)
**Number of Letters**	4.9 (1.3)	5.0 (1.2)	10.1 (1.7)
**Constituent-related semantic Transparency**	0.47 (0.13)	0.42 (0.10)	–

For the purpose of the lexical decision task, we further included a balanced set of 90 legal compound nonwords consisting of two embedded stem constituents [e.g., 6]. These were sub-divided into the exact same gender categories as the word targets (15 masculine-feminine, 15 masculine-neuter, 15 feminine-masculine, 15 feminine-neuter, 15 neuter-feminine, 15 neuter-masculine). It was ensured that the combination of the two embedded stems never formed a real word (e.g., *Seidenkeks* [silk biscuit]). Again, 30 compound nonword filler targets were included that included 10 masculine-masculine, 10 feminine-feminine and 10 neuter-neuter compound nonwords. The word and nonword targets were matched on length. The full list of materials is presented in Appendix A, and the English translations of the compound words and their constituents in Appendix B (see S1 File).

Every compound target was preceded by three different determiners (der_masc_, die_fem_, das_neut_), thus forming the following three priming conditions: head-congruent, modifier-congruent, and incongruent (see Table 1). Since the exact same target words were used in each of the three conditions, the target word properties were perfectly matched. To avoid that any participant saw any target more than once, we created three counterbalanced lists. Each list consisted of 240 trials (120 word targets, 120 nonword targets). The word trials consisted of 90 test items (where the determiner prime (der, die das) and the three conditions (head, modifier, incongruent) alternated and 30 filler items (again with alternating determiners (der, die, das) and only with congruent determiners). The same was true for the nonword trials, which consisted of 90 test items (with alternating primes and conditions) and 30 filler items. Following this, each trial was presented to 30 participants.

#### Procedure.

Each participant accessed the experiment online via Prolific (www.prolific.com), where they completed the primed lexical decision task via Gorilla Experiment Builder [72]. Participants were instructed to create a lab-like environment at home (i.e., participants were instructed to be in a quiet room with no other pet or person, with doors and windows closed, computers plugged in and mobile phones off). Numerous studies have shown now that valid response-time data can be collected in online-settings [e.g., 73–76]. A power estimate from GPower showed that a sample size of 90 participants was adequate to reach a power threshold of 80%, assuming an effect size of.3 [77]. Notably, we analyzed our data with LMEs instead of ANOVAs. Therefore, this power calculation can only be interpreted as an approximate heuristic. However, LMEs can model additional sources of variance and may therefore be more powerful than ANOVAs. In addition, we opted for a sample size that was comparable to prior primed lexical decision studies using morphologically complex words and nonwords [e.g., 78,79]. Participants were asked to answer as quickly and accurately as possible, and that they must decide whether the presented letter string was a real German word or not. Each trial began with presentation of a fixation cross for 1000 milliseconds (ms), followed by the presentation of the determiner prime (100 ms), followed by the target based on which participants made a lexical decision. There was a time limit of 3000 ms on the response, where the ‘M’ key was pushed with the right hand to response ‘YES’ to real German words and the ‘Z’ key was pushed with the left hand to respond ‘NO’ to a nonword. Trial presentation was randomized. Accuracy feedback was provided during eight practice trials (central green dot = correct, central red dot = incorrect). No accuracy feedback was provided during experimental trials. The experiment included three breaks. The whole experiment took approximately 13 minutes per participant.

### Results

Analyses were performed using generalised linear mixed-effects (LME) models [80] as implemented in the *lmer4* package [81] in the statistical software R version 4.3.3 [82]. As per pre-registration, fillers were excluded from the main analyses. Although the study’s focus was on compound words, compound nonwords were analysed separately, and are briefly reported below. Following Barr et al. (2013) [83], we determined the maximal random effect structure, which led us to include by-item and by-participant random intercepts, and by-item and by-participant random slopes for condition. LME models were produced via the *lmer* function for reaction times [RTs] and the *glmer* function for number of errors [ERs].

#### Preregistered main analyses.

**Fig 1:**
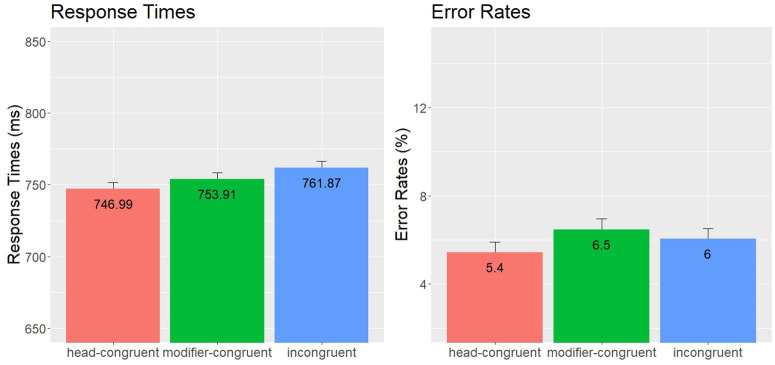
Compound Word Responses – Experiment 1: Mean Error Rates (%) and Lexical Decision Times (ms), including standard error bars.

Based on our preregistered data cleaning procedure, two participants were excluded from the analyses, because error rates were over 30%. Moreover, although not preregistered, six items with unusually high error rates (> 30%) were also removed from the analyses (i.e., *Maishuhn, Cellomusik, Pudelmütze, Holzkamm, Kürbiscurry, Schiffsrumpf*), which is in line with common data treatment standard in the field [41]. Incorrect responses were removed from the RT analysis (6.0% of responses), as were any responses made in less than 200 ms or after 2500 ms (0.14% of the data). A minimal model with random intercepts for participants and items only was fitted for the purpose of residual outlier trimming [80] which led to the removal of 2.0% of the data. A visual inspection of the raw RTs and a boxcox test determined that the raw RT data were not normally distributed. Based on results of the box cox test (λ = –1.27), an inverse transformation was applied to seek a more normal distribution of the dependent variables (−1000/RT). The maximal model contained factor condition (head-congruent, modifier-congruent, incongruent), TrialN, random intercepts for participants and items, as well as by-participant and by-item random slopes for factor condition (syntax of maximal linear mixed-effects model: DV ~ condition + scale(TrialN) + (1 + condition | participant) + (1 + condition | item). TrialN represented the order of trial presentation which was randomized for each participant, included to control for learning and fatigue effects. All continuous variables were scaled using the R function *scale* as part of the model-fitting procedure. The significance of the fixed effects in our LME models was determined with type III model comparisons using the *Anova* function in the *car* package [84]. The *emmeans* package was used to compute pairwise comparisons at the level of condition [85] which uses asymptotic z-tests with infinite denominator degrees of freedom. To correct for multiple comparisons, a Bonferroni correction was used to adjust the alpha significance level to 0.017 (0.05/3 = 0.017). The full LME outputs for all experiments are reported in the OSF repository of this study: https://osf.io/9mpn7/?view_only=959019ec6c644d2ca4976359daeced78. The mean ERs and RTs for the word conditions are reported in Fig 1.

In the RT analyses, there was a main effect of condition, *χ*2(2) = 9.67, *p* = .008, showing that participants responded faster in the head-congruent condition than in the incongruent condition (*z* = 3.04, *p* = .003), and marginally faster in the head-congruent condition than in the modifier-congruent condition (*z* = 1.99, *p* = .052). The difference between the modifier-congruent and incongruent conditions was not significant (*z* = 1.42, *p* = .160). There was also a significant effect of TrialN, *χ*2(1) = 16.90, *p* < .001. No other effects were significant. In the error analyses, there was also a main effect of condition, *χ*2(2) = 8.61, *p* = .014, showing that participants made fewer errors responding to words in the head-congruent condition than in the incongruent (*z* = 2.61, *p* = .009) and modifier-congruent conditions (*z* = 2.52, *p* = .012). The difference between the modifier-congruent and incongruent conditions was not significant (*z* = 0.32, *p* = .748). No other effects were significant.

#### Non-preregistered post-hoc analyses.

To test if the reported compound priming effects were modulated by item specific differences in semantic transparency, a variable that was not matched between head and modifier constituents, a set of (not pre-registered) post-hoc analyses were conducted. A (generalized) linear mixed-effects model was fitted for both RTs and error rates, with the fixed effects of condition (head-congruent, modifier-congruent, incongruent), head-frequency, modifier-frequency, head-transparency, modifier-transparency, the interaction between condition and head-transparency, the interaction between condition and modifier-transparency; TrialN, random intercepts for participants and items, and by-item and by-participant random slopes for factor condition. All continuous variables were scaled. In the RT analyses, there was a main effect of condition, *χ*2(2) = 9.49, *p* = .009, but the interactions between condition and head-transparency, and condition and modifier-transparency were not significant (*χ*2(2) = 0.20, *p* = .904; *χ*2(2) = 5.03, *p* = .081). The main effects of head-transparency and modifier-transparency were not significant (*χ*2(1) = 0.36, *p* = .549; *χ*2(1) = 0.001, *p* = .991). In the error analyses, there also was a main effect of condition, *χ*2(2) = 7.70, *p* = .021, but again, the interactions between condition and head-transparency, and condition and modifier-transparency were not significant (*χ*2(2) = 5.23, *p* = .073; *χ*2(2) = 2.00, *p* = .367). The main effects of head-transparency and modifier-transparency were not significant (*χ*2(1) = 1.61, *p* = .205; *χ*2(1) = 0.08, *p* = .772).

A second set of post-hoc analyses were conducted to control for differences in constituent gender and morpho-orthographic alternations on the observed priming effects. Compound words which included orthographic alternations of the embedded constituent morphemes are highlighted in the Appendix A with an asterisk (e.g., the linking suffix -n, as in *Tanne + Baum = Tannenbaum*; or the case of deletion-e’s, as in *Schule + Bus = Schulbus*). Since morpho-orthographic alternations were not restricted to compound words including feminine modifiers, but also occurred across other genders, all target words were coded for the presence (=1) or absence (=0) of orthographic alternations of the embedded stem constituents. A (generalized) linear mixed-effects model was fitted for both RTs and error rates, with the fixed effects of condition (head-congruent, modifier-congruent, incongruent), gender (f-m, f-n, f-f, m-f, m-n, m-m, n-f, n-m, n-n), orthographic alternation (present = 1, absent = 0), TrialN, and two random effects factors (random intercepts and random slopes for subjects and items). In the RT analyses, there was a main effect of condition, *χ*2(2) = 9.24, *p* = .010, but the main effect of gender (*χ*2(1) = 2.49, *p* = .778) and the main effect of orthographic alternation were not significant (*χ*2(1) = 0.32, *p* = .572). In the error analyses, there was a main effect of condition, *χ*2(2) = 8.17, *p* = .012. No other effects were significant. These results suggest that morpho-orthographic alternations of the feminine modifier constituents did not modulate the observed priming effects, which is consistent with prior findings [86].

**Fig 2:**
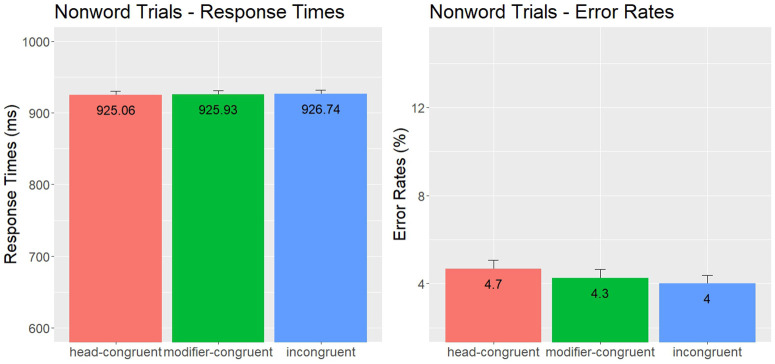
Compound Nonword Responses – Experiment 1: Mean Error Rates (%) and Lexical Decision Times (ms), including standard error bars.

***Nonword analyses*:** RT data were inverse transformed. The final model contained the factors condition (head-congruent, modifier-congruent, incongruent), TrialN, head constituent frequency, modifier constituent frequency, as well as random intercepts and random slopes for subjects and items, and by-item and by-participant random slopes for the factor condition. All continuous variables were scaled. The mean RTs and error rates are presented in Fig 2. In the RT analyses, there were no significant effects. In the error analyses, there was a main effect of head constituent frequency (*χ*2(1) = 7.70, *p* = .006) and modifier constituent frequency (*χ*2(1) = 4.53, *p* = .033), showing that the higher the constituent frequency, the harder it was to reject the nonword. No other effects were significant.

### Discussion

The results of Experiment 1 showed that determiner primes that were gender-congruent with the head constituent (and, thus, the compound as a whole) facilitated compound word recognition relative to the incongruent control, replicating priming effects with morphologically simple nouns, reported in previous lexical decision studies [for a review, see 49]. These findings are also consistent with prior studies showing that lexical decisions to nouns are slowed when preceded by gender-incongruent determiner or adjective primes [e.g., 59,60]. Post-hoc analyses confirmed that these priming effects remained robust when semantic constituent transparency, constituent gender, and morpho-orthographic alternations were accounted for within the analyses. No evidence for modifier-congruent priming effects were observed. We reasoned that the absence of modifier-priming effects may have been due to the relatively short prime presentation durations (i.e., 100ms) in Experiment 1. It has been shown that syntactic and semantic aspects of morphological processing are closely intertwined, and that syntactic/semantic influences on lexical decision tend to increase when participants are given more time to thoroughly process the prime [e.g., 87,88]. To provide more opportunity for modifier-priming effects to arise during compound word recognition, we conducted a second experiment with a new participant sample, using a slightly longer prime duration (200ms).

## Experiment 2

### Method

#### Participants.

Ninety adult participants (35 females; age: *M* = 29 years, *SD* = 6.8, min = 20 years, max = 47 years) were recruited from within Germany using Prolific (www.prolific.co). All participants were monolingual native German speakers, residing in Germany, with no learning impairments or history of neurological impairment. They were paid at a rate of £11/hour for their time. Experiment 2 was conducted between December 2022 and January 2023. All participants have given their consent to participate.

#### Materials.

The materials were identical to Experiment 1.

#### Procedure.

The procedure was identical to Experiment 1, except that determiner primes were presented for 200 ms (100 ms longer than in Experiment 1).

### Results

Analyses were performed using the same principles as in Experiment 1.

#### Preregistered main analyses.

**Fig 3:**
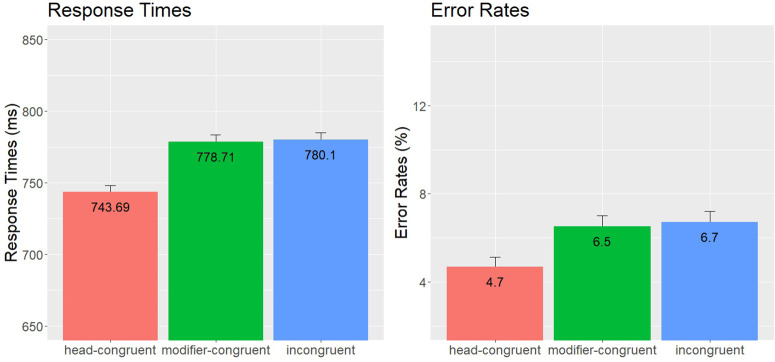
Compound Word Responses – Experiment 2: Mean Error Rates (%) and Lexical Decision Times (ms), including standard error bars.

Nonword fillers were removed from the main analysis. Although not preregistered, seven items with unusually high error rates (> 30%) were also removed from the analyses (i.e., *Maishuhn, Cellomusik, Pudelmütze, Holzkamm, Kürbiscurry, Kartoffelacker, Stockente*) and five participants were excluded from the analyses, because error rates were over 30%. Incorrect responses were removed from the RT analysis (5.6% of responses), as were any responses made in less than 200 ms or after 2500 ms (0.17% of the data). A minimal model for residual trimming of outliers was used [80] which led to the removal of 1.6% of the data. A visual inspection of the raw RTs and a boxcox test determined that the raw RT data were not normally distributed. Based on results of the box cox test (λ = –0.3), an inverse transformation was applied to seek a more normal distribution of the dependent variables. Exactly like in Experiment 1, the maximal model contained factor condition (head-congruent, modifier-congruent, incongruent), TrialN, random intercepts for participants and items, as well as by-participant and by-item random slopes for factor condition. All continuous variables were scaled. Mean ERs and RTs for the word conditions are reported in Fig 3.

In the RT analyses, there was a main effect of condition, *χ*2(2) = 84.67, *p* < .001, showing that participants responded faster in the head-congruent condition than in the incongruent (*z* = 7.90, *p* < .001) and modifier-congruent conditions (*z* = 8.17, *p* < .001). However, the difference between the modifier-congruent and incongruent conditions was not significant (*z* = 0.15, *p* = .883). There was also a significant effect of TrialN, *χ*2(1) = 41.26, *p* < .001. No other effects were significant. In the error analyses, there was also a main effect of condition, *χ*2(2) = 19.78, *p* < .001, showing that made fewer errors responding to words in the head-congruent condition than in the incongruent (*z* = 3.29, *p* = .001) and modifier-congruent conditions (*z* = 4.33, *p* < .001). The difference between the modifier-congruent and incongruent condition was not significant (*z* = 1.21, *p* = .227). There was also a significant main effect of TrialN, *χ*2(1) = 4.94, *p* = .026. No other effects were significant. The results of Experiment 2 thus replicate the findings reported in Experiment 1, suggesting that head-congruent, but not modifier-congruent determiner primes facilitate the visual recognition of compound words. This implies that even when participants had more time to more thoroughly process the primes (with 200 ms prime presentation durations in Experiment 2), the modifier effects did not emerge.

#### Non-preregistered post-hoc analyses.

**Fig 4:**
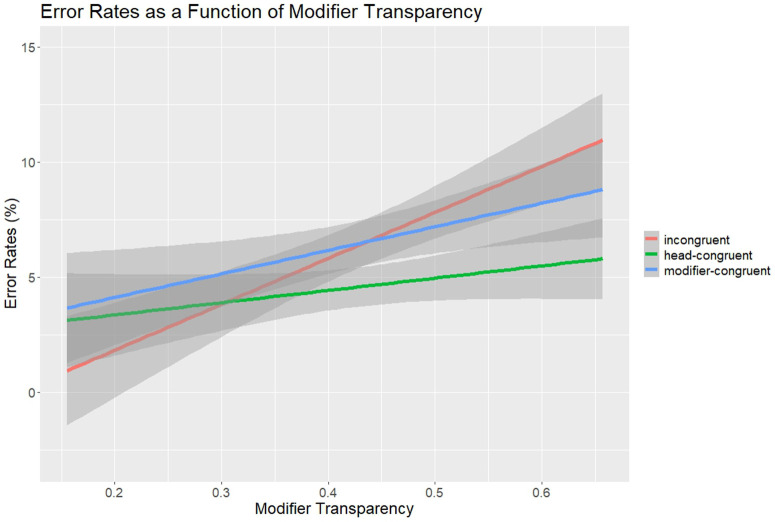
Experiment 2: Error Rates as a Function of Modifier Transparency.

The same post-hoc analyses as in Experiment 1 were conducted to test if the reported compound priming effects were modulated by item specific differences in semantic transparency. A (generalized) linear mixed-effects model was fitted for both RTs and error rates, with the fixed effects of condition (head-congruent, modifier-congruent, incongruent), head-transparency, modifier-transparency, the interaction between condition and head-transparency, the interaction between condition and modifier-transparency; TrialN, and two random effects factors (random intercepts and random slopes for subjects and items). In the RT analyses, there was a main effect of condition, *χ*2(2) = 71.66, *p* < .001, but the interactions between condition and head-transparency, and condition and modifier-transparency were not significant (*χ*2(1) = 0.72, *p* = .698; *χ*2(1) = 0.46, *p* = .793). The main effects of head-transparency and modifier-transparency were not significant (*χ*2(1) = 0.02, *p* = .894; *χ*2(1) = 1.34, *p* = .247). There was also a significant effect of TrialN, *χ*2(1) = 40.34, *p* < .001. No other effects were significant. In the error analyses, there also was a main effect of condition, *χ*2(2) = 16.35, *p* < .001, and a significant interaction between condition and modifier-transparency, *χ*2(2) = 6.79, *p* = .034, suggesting that both head and modifier priming effects increased with increasing modifier transparency (see Fig 4). The interaction between condition and head-transparency was not significant, *χ*2(2) = 2.65, *p* = .266. The main effects of head-transparency and modifier-transparency were not significant (*χ*2(1) = 0.25, *p* = .615; *χ*2(1) = 2.01, *p* = .156). No other effects were significant.

As in Experiment 1, a second set of post-hoc analyses were conducted to control for differences in constituent gender and orthographic alternations on the observed priming effects. A (generalized) linear mixed-effects model was fitted for both RTs and error rates, with the fixed effects of condition (head-congruent, modifier-congruent, incongruent), gender (f-m, f-n, f-f, m-f, m-n, m-m, n-f, n-m, n-n), orthographic alternation (0, 1), TrialN, and two random effects factors (random intercepts and random slopes for subjects and items). In the RT analyses, there was a main effect of condition, *χ*2(2) = 73.02, *p* < .001, but the main effect of gender (*χ*2(5) = 2.62, *p* = .758) and the main effect of orthographic alternation were not significant (*χ*2(5) = 2.52, *p* = .112). There was also a significant effect of TrialN, *χ*2(1) = 40.72, *p* < .001. No other effects were significant. In the error analyses, there was a main effect of condition, *χ*2(2) = 14.57, *p* < .001, but the main effect of gender (*χ*2(5) = 2.50, *p* = .776) and the main effect of orthographic alternation were not significant (*χ*2(1) = 1.41, *p* = .235). No other effects were significant.

**Fig 5:**
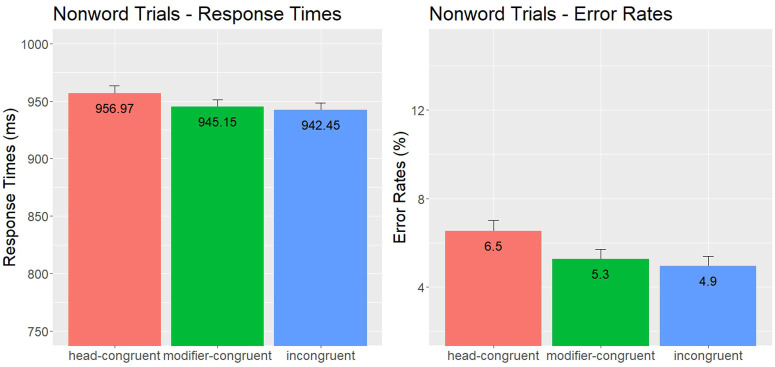
Compound Nonword Responses – Experiment 2: Mean Error Rates (%) and Lexical Decision Times (ms), including standard error bars.

***Nonword analyses*:** RT data were inverse transformed. The final model contained factor condition (head-congruent, modifier-congruent, incongruent), TrialN, head constituent frequency, modifier constituent frequency, random intercepts and random slopes for subjects and items, and by-item random slopes for factor condition. All continuous variables were scaled. The mean RTs and error rates are presented in Fig 5. In the RT analyses, there were no significant effects, although the mean RTs showed that, on average, nonwords in the head-congruent condition were rejected more slowly than the items in the other two conditions. In the error analyses, there was a main effect of head constituent frequency (*χ*2(1) = 6.50, *p* = .011), showing that the higher the head constituent frequency, the harder it was to reject the nonword. No other effects were significant.

### Discussion

Similar to Experiment 1, the head-congruent determiner primes produced priming, whereas modifier-congruent determiners did not. The post-hoc analyses confirmed that determiner-compound priming effects remained robust when semantic constituent transparency, constituent gender and the presence of interfixes were accounted for within the analyses.

## General discussion

The present study tested how German compound nouns and their grammatical gender are lexically stored and processed during visual word recognition. We used a visual lexical decision task with German noun-noun compound targets that were preceded by gender-marked determiner primes. Determiner primes were either gender-congruent with the compounds’ head, their modifier or completely incongruent. According to current accounts, morphologically complex words, including compound words, are rapidly decomposed into morphemic subunits during visual word recognition [e.g., 28,42,89]. Empirical data suggest that both whole words and morphemes are accessed in parallel at the level of the orthographic lexicon [37,38]. Thus, a given compound word will activate its embedded morphemic constituents (e.g., *water* and *bottle*) in addition to the whole word (*waterbottle*). Following this, the morpheme representations at the word-form level should automatically activate the corresponding syntactic (lemma) representations. Thus, morpheme-based syntactic effects were expected here. Furthermore, there are empirical data from both syntactic and semantic effects in compound comprehension and production (picture naming), suggesting constituent activation at lemma level, in line with a multiple-lemma representation of German compounds (for comprehension, see [13,22]; for derived words, see [90]; for production, see [21,24]; but see [16,17,26]). We predicted modifier-related priming effects in visual compound recognition, in addition to head-priming effects. This, however, was not confirmed in our data. Instead, head-priming effects were observed exclusively, whereas no significant effects were obtained for the modifier condition. Participants were faster to decide that a letter string was an existing word of German when the determiner prime was gender-congruent with the morphological head and, thus, the whole word when compared with incongruent determiner primes. In contrast, determiner primes that were congruent with the modifier constituent of the compound target did not affect compound word recognition latencies.

Various post-hoc analyses revealed that the determiner-priming effects were not modulated by grammatical gender of compound targets or by morpho-orthographic alternations of the modifier constituent, such as the existence of interfixes (e.g., *Pferd-e-schwanz* [ponytail]). This was important to check because the existence of an interfix may have complicated visual compound recognition, decomposition and priming effects that are related to the first (modifier) constituent of compound targets [91].

Notably, the observed head-priming effects during compound recognition might have different functional origins. First, a facilitatory priming effect at lemma level would result in faster responses with head-congruent but incongruent determiner primes (e.g., for gender priming in determiner-primed production [34,64]). Second, the observed head-congruency effects might follow from an inhibitory effect during post-lexical checking, thus, resulting in slower responses in the case of incongruent determiner primes (e.g., [34]). Because we did not include a gender-neutral control condition, it is not possible to disentangle between these two explanations (see also [24]).

In contrast to the observed head-congruency effects, no significant effects were obtained in the modifier-related condition. One explanation might be that the modifier’s gender and determiner are not activated due to the holistic activation of compounds at the lemma level (for speech production, [46]). Empirical data, however, suggest a multiple-lemma representation of compounds [e.g., 21,22,24]. Notably, there are at least two other explanations for the absence of any effects in the modifier-congruent condition in the lexical-decision task. First, the observed null effect in the modifier-related condition may follow from a trade-off between facilitation and inhibition of modifier-congruent but overall incongruent determiner primes. In other words, while the modifier-congruent determiner primes might have facilitated the activation of the modifier constituent of compound targets at lemma level, they might have inhibited the activation of the head constituent and, thus, the whole compound at a post-lexical level of processing. Thus, the simultaneous facilitation and inhibition may have cancelled each other out [24]. Another explanation for the absence of modifier priming effects is that, although the syntactic properties of the whole-compound and its constituents were activated, the processing of the syntactic properties of the whole-compound were prioritised in this task, due to the lexical decision made at the level of the whole-word.

Our study used visual lexical decision with unmasked priming to test how compound nouns are lexically stored and processed during visual compound recognition. The unmasked presentation of gender-marked determiner primes may have allowed the participants to use a head-based strategy, that is to concentrate more on the last constituent, the compound’s head. The head determines lexical syntax of the compound (e.g., word class, grammatical gender), whereas the modifier can differ in syntactic properties. Following this, even in the case of constituent-based processing, head-related syntactic effects are likely stronger than modifier-related effects [24,26]. Furthermore, the lexical decision task is known to include a post-lexical meta-linguistic decision component. This may delay and cover experimental effects [e.g., 93]. It has been previously shown that abstract gender features of determiner primes and target nouns are automatically activated during visual word recognition, even though not necessarily needed for the task [e.g., 33,35; for production, 34]. As such, the repeated presentation of determiner primes in close succession with a noun may automatically result in the internal activation of determiner-noun phrases [see 24 for discussion of primed picture-naming data]. While determiner forms are stored at word-form level of the mental lexicon (*der, die, das* [the]), they are linked to their gender features (masculine, feminine, neuter). Thus, the activation of abstract lexical-syntactic information (gender) is needed for the (internal) activation of determiner-noun phrases. Following this, both the lemma level and the word-form level are likely involved in the primed lexical decision task, but there may be less scope for morpho-syntactic constituent processing of compounds. This notion is also consistent with recent findings by Rossetto and colleagues [78], who carried out a lexical decision task with affixed nonwords and found that the syntactic constraints of the embedded morphemic constituents did not affect affixed nonword processing (e.g., *proudment*). Their findings thus further support the idea that constituent-specific syntactic processing may have comparatively little impact on participants’ performance in the lexical decision task [for discussion in line with morphological transcendence, see 92,94,95].

Our findings are inconsistent with the ERP data reported by Koester and colleagues (2004), investigating the auditory processing of determiner-compound noun phrases in German [22]. In this EEG-study, determiners were either congruent or incongruent with the first or second constituent of noun-noun compound targets. To ensure that participants pay attention to the determiners and compound targets a grammaticality judgement task (gender agreement) and a semantic decision task were implemented. Both modifier- and head-incongruency of determiner and constituent elicited a left-anterior negativity (LAN), thus indicating constituent-specific activation of grammatical gender during the auditory recognition of determiner-compound noun phrases. In previous studies, LAN effects have been reported for syntactic and morphological violations [e.g., 96,97]. The data, thus, implies the separate retrieval of a compound’s constituents at lemma level. However, during auditory processing, the speech signal unfolds over time that might induce a constituent-specific processing mechanism during auditory compound comprehension. This is likely different in visual compound processing: While reading proceeds from left to right, eye tracking data suggest that during compound reading the whole word is available from the first fixation, already [e.g., 12,15].

In exploratory analyses, we also checked whether determiner-priming effects were modulated by the semantic transparency of the compound targets. This seemed to be important because other studies showed that the type of processing and lexical representation of compounds may be influenced by the semantic transparency of the underlying constituents of compounds [e.g., 21; see also 6,98,100]. The error analysis of Experiment 2 revealed a significant interaction between priming condition and modifier transparency: Both head- and modifier-priming effects increased with increasing modifier transparency, whereas the transparency of the morphological head did not modulate the priming effects. Thus, the activation of a compound and its constituents was graded by the modifier’s semantic transparency, in line with dual-route or gradient models of morphological processing, predicting stronger constituent-specific effects with higher semantic transparency [e.g., 98–100]. In a continuous picture naming study with noun-noun compound targets, the modifier-related semantic effects were also modulated by the semantic transparency of compound targets, suggesting an influence of the semantic transparency of the morphological head at lemma level [21].

Notably, no semantic transparency effects were observed in Experiment 1, when a shorter prime duration was used (100 ms), and no transparency effects were observed in the reaction time analysis, indicating a relatively weak impact of semantic transparency in this task. Furthermore, it is not clear why the semantic transparency of the morphological head did not modulate the effects. However, semantic transparency was not perfectly balanced between modifier and head in our materials. On average, modifier-transparency was lower than head-transparency (see Table 2), suggesting that the meaning of the modifiers deviated more clearly from the meaning of the whole compound, which may have provided more opportunity for modifier-transparency effects to arise.

Furthermore, we asked whether modifier-congruency effects would be present in the case of *novel* compounds. Since novel compounds don’t have a lexical entry, we predicted to observe modifier-related effects here [see 26, Exp.3, for determiner priming effects in novel compound production]. The results revealed no significant determiner-priming effects with novel compounds, that is neither head- nor modifier-congruent primes modulated the responses for novel compounds However, in the error analyses, there was a main effect of head constituent frequency, showing that the higher the head constituent frequency, the harder it was to reject the nonword. In contrast, no significant modifier frequency effects were obtained. This may indicate that participants concentrated more on the head than the modifier of (novel) compound targets during the lexical decision task. In sum, even for novel compounds that are not lexicalized, reliance on the morphological head seemed to be stronger.

This study mainly focused on two competing accounts explaining how compounds may be represented at a lexical-syntactic level of the mental lexicon (single- vs. multiple lemma model; [46] vs. [25]). However, the empirical data from this and other studies did not clearly point to one or the other model, which may be linked to the hybrid nature of the multiple-lemma model of compounds that includes a compound lemma representation in addition to the corresponding constituent lemmas [25; see also 24,26]. Furthermore, recent studies show that the processing of compounds is determined by various factors, including role-dependent meaning representations of embedded constituents of compounds, their semantic transparency and constituent and compound frequency [e.g., 95,100].

### Limitations and future directions

A picture that emerges from the literature is that head constituents clearly play a dominant role in compound word recognition and production (for data from aphasia, see [101]). Head-related determiner-priming effects were obtained in all studies, testing compounds in comprehension or production, whereas modifier-related effects are often weak or completely absent [e.g., 24,26]. However, there are methodological considerations that potentially modulate the extent to which participants rely on the modifier constituent in compound word processing. The presentation of determiner primes in a lexical decision task might induce a head-related processing strategy, involving a stronger reliance on the second than the first constituent during compound processing in German [24]. This in turn may lead to a reduction in modifier-related priming effects, for both familiar and novel compounds, even under the assumption of a multiple-lemma representation for compound words. Notably, while this processing strategy may be successful in processing of right-headed German compounds, a different strategy would be needed for compound recognition in other languages, such as Italian or Greek, that include both left- and right-headed compounds [e.g., 102,103].

A further methodological consideration for future research is the presentation duration of the determiner primes. The overt presentation of determiner primes in the present lexical decision task may have induced strategic processes and a task-related emphasis on the compounds’ head constituents. One option to reduce participants’ reliance on the morphological head of compounds may be an extension of the current study to the masked priming paradigm using very brief and masked 50 ms primes. If the here-reported head-priming effects were indeed due to strategic decision-making processes, the masking of the determiner primes may provide more opportunity to capture the early lexical-syntactic activation of the embedded morphemic constituents, including the modifier constituent. Furthermore, the implementation of monomorphemic filler words may help to avoid head-related processing strategies during compound word recognition. Another tool to capture processes that are too transient to impact behavioral response data, would be the measurement of event-related potentials (ERPs) whilst administering the determiner-compound priming task.

It is also worth noting that online experiments offer less control over the testing environment than laboratory-based research. In particular, hardware characteristics such as monitor refresh rate, screen size, luminance, and viewing distance cannot be standardised across participants and may introduce additional variability into the data. While these limitations are inherent to remote testing, appropriate design choices and statistical approaches can help mitigate their impact and ensure that key findings remain robust. To further test the generalisability of our findings, a replication of the current study in a controlled laboratory setting would provide a more standardised test of the current hypotheses.

Finally, given that compound processing effects have been found to change with age, it may be of interest for future research to further explore age-related changes in the comprehension and production of morphologically complex words [e.g., 27,104], keeping in mind that the current findings from compound words may fundamentally differ from other forms of morphological complexity including affixation (for grammatical gender effects in derived words, see [90]). Our visual compound recognition data from young adult readers partially converge with the compound production data by Lorenz and colleagues who tested young adult speakers [26]. They presented determiner primes in a picture naming task with noun-noun compound targets and reported head-priming effects but no modifier-priming effects. Critically however, more robust modifier-congruent determiner-priming effects have been observed in participants with aphasia and older language-unimpaired speakers [24], suggesting that lexical-syntactic processing of constituents may become more critical as we age.

## Conclusions

A visual lexical decision task was used to test determiner-priming effects related to constituents of noun-noun compound targets in German. Determiner primes were either congruent with the compound’s head and, thus, the compound as a whole, or they were congruent with the compound’s modifier. Both conditions were compared to a gender-incongruent determiner condition. While head-priming effects were significant in both experiments, no modifier-priming effects were observed in the reaction-time data. The data suggest activation of the compound’s/head’s gender, whereas gender properties of non-head constituents did not modulate participants’ responses in the lexical decision task. The semantic transparency of non-head constituents modulated both head- and modifier-priming effects in response accuracies, suggesting a role of semantic transparency in the constituent-specific activation during compound word recognition. Our findings support the notion that head constituents play a dominant role in the visual recognition of compound words at a lexical-syntactic level.

## Supporting information

S1 File Materials. (PDF)
