# Supplementary material for: Head-driven gender assignment in noun-noun compound recognition: Evidence from a determiner priming task in German
Source: PLoS One. 2026 May 20;21(5):e0348377. doi: 10.1371/journal.pone.0348377 (PMC13189298; doi:10.1371/journal.pone.0348377)
Supplement: S1 File — (PDF) [file pone.0348377.s001.pdf]

Supporting information: S1 materials

Appendix A

Table A1: Word and nonword trials and determiner primes used in Experiments 1 and 2.

Compound Target	Gender	Type	Modifier	Head	Head Congr	Mod Congr	Incongr Control
Mülltonne	**m-f**	target	Müll	Tonne	die	der	das
Schnabeltasse	**m-f**	target	Schnabel	Tasse	die	der	das
Windmühle	**m-f**	target	Wind	Mühle	die	der	das
Kaffeemaschine	**m-f**	target	Kaffee	Maschine	die	der	das
Stockente	**m-f**	target	Stock	Ente	die	der	das
Nagelfeile	**m-f**	target	Nagel	Feile	die	der	das
Käsetheke	**m-f**	target	Käse	Theke	die	der	das
Sanduhr	**m-f**	target	Sand	Uhr	die	der	das
Briefmarke	**m-f**	target	Brief	Marke	die	der	das
Stachelbeere	**m-f**	target	Stachel	Beere	die	der	das
Latzhose	**m-f**	target	Latz	Hose	die	der	das
Hundehütte*	**m-f**	target	Hund	Hütte	die	der	das
Pudelmütze	**m-f**	target	Pudel	Mütze	die	der	das
Kreissäge	**m-f**	target	Kreis	Säge	die	der	das
Rauchwolke	**m-f**	target	Rauch	Wolke	die	der	das
Eselsohr*	**m-n**	target	Esel	Ohr	das	der	die
Spiegelei	**m-n**	target	Spiegel	Ei	das	der	die
Schlauchboot	**m-n**	target	Schlauch	Boot	das	der	die
Kleeblatt	**m-n**	target	Klee	Blatt	das	der	die
Armband	**m-n**	target	Arm	Band	das	der	die
Zirkuszelt	**m-n**	target	Zirkus	Zelt	das	der	die
Tischbein	**m-n**	target	Tisch	Bein	das	der	die
Gürteltier	**m-n**	target	Gürtel	Tier	das	der	die
Chorkonzert	**m-n**	target	Chor	Konzert	das	der	die
Stromkabel	**m-n**	target	Strom	Kabel	das	der	die
Teelicht	**m-n**	target	Tee	Licht	das	der	die
Maishuhn	**m-n**	target	Mais	Huhn	das	der	die
Halstuch	**m-n**	target	Hals	Tuch	das	der	die
Kürbiscurry	**m-n**	target	Kürbis	Curry	das	der	die
Riesenrad*	**m-n**	target	Riese	Rad	das	der	die
Rosenbusch*	**f-m**	target	Rose	Busch	der	die	das
Blumentopf*	**f-m**	target	Blume	Topf	der	die	das
Geigenkasten*	**f-m**	target	Geige	Kasten	der	die	das
Lippenstift*	**f-m**	target	Lippe	Stift	der	die	das
Kassenbon*	**f-m**	target	Kasse	Bon	der	die	das
Kirchturm*	**f-m**	target	Kirche	Turm	der	die	das
Federball	**f-m**	target	Feder	Ball	der	die	das
Sonnenschirm*	**f-m**	target	Sonne	Schirm	der	die	das
Handschuh	**f-m**	target	Hand	Schuh	der	die	das

Aktenschrank*	**f-m**	target	Akte	Schrank	der	die	das
Kartoffelacker	**f-m**	target	Kartoffel	Acker	der	die	das
Tannenbaum*	**f-m**	target	Tanne	Baum	der	die	das
Schulbus*	**f-m**	target	Schule	Bus	der	die	das
Bienenhonig*	**f-m**	target	Biene	Honig	der	die	das
Fliegenpilz*	**f-m**	target	Fliege	Pilz	der	die	das
Butterbrot	**f-n**	target	Butter	Brot	das	die	der
Tintenfass*	**f-n**	target	Tinte	Fass	das	die	der
Wespennest*	**f-n**	target	Wespe	Nest	das	die	der
Kunsthaar	**f-n**	target	Kunst	Haar	das	die	der
Spinnennetz*	**f-n**	target	Spinne	Netz	das	die	der
Polizeiauto	**f-n**	target	Polizei	Auto	das	die	der
Nudelsieb	**f-n**	target	Nudel	Sieb	das	die	der
Prachtstück	**f-n**	target	Pracht	Stück	das	die	der
Wurzelgemüse	**f-n**	target	Wurzel	Gemüse	das	die	der
Jagdgewehr	**f-n**	target	Jagd	Gewehr	das	die	der
Schneckenhaus*	**f-n**	target	Schnecke	Haus	das	die	der
Brautkleid	**f-n**	target	Braut	Kleid	das	die	der
Übungsheft*	**f-n**	target	Übung	Heft	das	die	der
Damenparfüm*	**f-n**	target	Dame	Parfüm	das	die	der
Olivenöl*	**f-n**	target	Olive	Öl	das	die	der
Pferdeschwanz*	**n-m**	target	Pferd	Schwanz	der	das	die
Goldfisch	**n-m**	target	Gold	Fisch	der	das	die
Geldschein	**n-m**	target	Geld	Schein	der	das	die
Eisbär	**n-m**	target	Eis	Bär	der	das	die
Tennisprofi	**n-m**	target	Tennis	Profi	der	das	die
Grashalm	**n-m**	target	Gras	Halm	der	das	die
Fleischwolf	**n-m**	target	Fleisch	Wolf	der	das	die
Papierkorb	**n-m**	target	Papier	Korb	der	das	die
Schiffsrumpf*	**n-m**	target	Schiff	Rumpf	der	das	die
Rohrzucker	**n-m**	target	Rohr	Zucker	der	das	die
Benzinkanister	**n-m**	target	Benzin	Kanister	der	das	die
Feldhase	**n-m**	target	Feld	Hase	der	das	die
Glasteller	**n-m**	target	Glas	Teller	der	das	die
Holzkamm	**n-m**	target	Holz	Kamm	der	das	die
Kinofilm	**n-m**	target	Kino	Film	der	das	die
Cellomusik	**n-f**	target	Cello	Musik	die	das	der
Billiardkugel	**n-f**	target	Billiard	Kugel	die	das	der
Gasmaske	**n-f**	target	Gas	Maske	die	das	der
Büroklammer	**n-f**	target	Büro	Klammer	die	das	der
Silbermünze	**n-f**	target	Silber	Münze	die	das	der
Telefonzelle	**n-f**	target	Telefon	Zelle	die	das	der
Schildkröte	**n-f**	target	Schild	Kröte	die	das	der
Bierflasche	**n-f**	target	Bier	Flasche	die	das	der
Landkarte	**n-f**	target	Land	Karte	die	das	der
Wasserwaage	**n-f**	target	Wasser	Waage	die	das	der
Kanutour	**n-f**	target	Kanu	Tour	die	das	der
Seilbahn	**n-f**	target	Seil	Bahn	die	das	der

Wunderkerze	**n-f**	target	Wunder	Kerze	die	das	der
Augenklappe*	**n-f**	target	Auge	Klappe	die	das	der
Klobrille	**n-f**	target	Klo	Brille	die	das	der
Gartenzwerg	**m-m**	filler	Garten	Zwerg	der	der	der
Wintermantel	**m-m**	filler	Winter	Mantel	der	der	der
Bambusstab	**m-m**	filler	Bambus	Stab	der	der	der
Regenbogen	**m-m**	filler	Regen	Bogen	der	der	der
Apfelkuchen	**m-m**	filler	Apfel	Kuchen	der	der	der
Kofferraum	**m-m**	filler	Koffer	Raum	der	der	der
Hammerhai	**m-m**	filler	Hammer	Hai	der	der	der
Hirschkäfer	**m-m**	filler	Hirsch	Käfer	der	der	der
Löwenzahn*	**m-m**	filler	Löwe	Zahn	der	der	der
Seeigel	**m-m**	filler	See	Igel	der	der	der
Schafsfell*	**n-n**	filler	Schaf	Fell	das	das	das
Dachfenster	**n-n**	filler	Dach	Fenster	das	das	das
Feuerzeug	**n-n**	filler	Feuer	Zeug	das	das	das
Bilderbuch*	**n-n**	filler	Bild	Buch	das	das	das
Fotoalbum	**n-n**	filler	Foto	Album	das	das	das
Kinderbett*	**n-n**	filler	Kind	Bett	das	das	das
Meerschwein	**n-n**	filler	Meer	Schwein	das	das	das
Blutgerinnsel	**n-n**	filler	Blut	Gerinnsel	das	das	das
Dorffest	**n-n**	filler	Dorf	Fest	das	das	das
Fieberthermometer	**n-n**	filler	Fieber	Thermome ter	das	das	das
Suppenkelle*	**f-f**	filler	Suppe	Kelle	die	die	die
Türklinke	**f-f**	filler	Tür	Klinke	die	die	die
Heckenschere*	**f-f**	filler	Hecke	Schere	die	die	die
Mausefalle*	**f-f**	filler	Maus	Falle	die	die	die
Seifenblase*	**f-f**	filler	Seife	Blase	die	die	die
Kuhweide	**f-f**	filler	Kuh	Weide	die	die	die
Luftpumpe	**f-f**	filler	Luft	Pumpe	die	die	die
Leberwurst	**f-f**	filler	Leber	Wurst	die	die	die
Taschenlampe*	**f-f**	filler	Tasche	Lampe	die	die	die
Milchstrasse	**f-f**	filler	Milch	Straße	die	die	die
Pelzampel	**m-f**	nonword	Pelz	Ampel	die	der	das
Vulkanechse	**m-f**	nonword	Vulkan	Echse	die	der	das
Heldlaus	**m-f**	nonword	Held	Laus	die	der	das
Würfelratte	**m-f**	nonword	Würfel	Ratte	die	der	das
Breizwiebel	**m-f**	nonword	Brei	Zwiebel	die	der	das
Hutschraube	**m-f**	nonword	Hut	Schraube	die	der	das
Kernrassel	**m-f**	nonword	Kern	Rassel	die	der	das
Ingwerflöte	**m-f**	nonword	Ingwer	Flöte	die	der	das
Geizpfote	**m-f**	nonword	Geiz	Pfote	die	der	das
Kamindüne	**m-f**	nonword	Kamin	Düne	die	der	das
Clubstadt	**m-f**	nonword	Club	Stadt	die	der	das
Keimeule	**m-f**	nonword	Keim	Eule	die	der	das
Dieblinse	**m-f**	nonword	Dieb	Linse	die	der	das
Duftspritze	**m-f**	nonword	Duft	Spritze	die	der	das

Pudergondel	**m-f**	nonword	Puder	Gondel	die	der	das
Ochsenbanjo*	**m-n**	nonword	Ochse	Banjo	das	der	die
Löffelknie	**m-n**	nonword	Löffel	Knie	das	der	die
Himmelsiglu*	**m-n**	nonword	Himmel	Iglu	das	der	die
Pinguinfloß	**m-n**	nonword	Pinguin	Floß	das	der	die
Dornschwert	**m-n**	nonword	Dorn	Schwert	das	der	die
Flügelbaby	**m-n**	nonword	Flügel	Baby	das	der	die
Zehenstadion	**m-n**	nonword	Zehen	Stadion	das	der	die
Darmkinn	**m-n**	nonword	Darm	Kinn	das	der	die
Teppichbeil	**m-n**	nonword	Teppich	Beil	das	der	die
Weizensalz	**m-n**	nonword	Weizen	Salz	das	der	die
Walddeck	**m-n**	nonword	Wald	Deck	das	der	die
Käfigherz	**m-n**	nonword	Käfig	Herz	das	der	die
Rabenlamm*	**m-n**	nonword	Rabe	Lamm	das	der	die
Spinatreh	**m-n**	nonword	Spinat	Reh	das	der	die
Gangrecht	**m-n**	nonword	Gang	Recht	das	der	die
Brezelfuß	**f-m**	nonword	Brezel	Fuß	der	die	das
Bürstenstein*	**f-m**	nonword	Bürste	Stein	der	die	das
Traubenfilz*	**f-m**	nonword	Traube	Filz	der	die	das
Muschelaffe	**f-m**	nonword	Muschel	Affe	der	die	das
Katzenlauch*	**f-m**	nonword	Katze	Lauch	der	die	das
Raupenhering*	**f-m**	nonword	Raupe	Hering	der	die	das
Orgelsekt	**f-m**	nonword	Orgel	Sekt	der	die	das
Zangenfasan*	**f-m**	nonword	Zange	Fasan	der	die	das
Vasengreis*	**f-m**	nonword	Vase	Greis	der	die	das
Waffelfuchs	**f-m**	nonword	Waffel	Fuchs	der	die	das
Mauerhelm	**f-m**	nonword	Mauer	Helm	der	die	das
Tulpenfink*	**f-m**	nonword	Tulpe	Fink	der	die	das
Fackelfarn	**f-m**	nonword	Fackel	Farn	der	die	das
Seidenkeks*	**f-m**	nonword	Seide	Keks	der	die	das
Taubenmotor*	**f-m**	nonword	Taube	Motor	der	die	das
Nadeltrikot	**f-n**	nonword	Nadel	Trikot	das	die	der
Nasentandem*	**f-n**	nonword	Nase	Tandem	das	die	der
Flutgewächs	**f-n**	nonword	Flut	Gewächs	das	die	der
Notensofa*	**f-n**	nonword	Note	Sofa	das	die	der
Tütentaxi*	**f-n**	nonword	Tüte	Taxi	das	die	der
Birnenmetall*	**f-n**	nonword	Birne	Metall	das	die	der
Pfannentor*	**f-n**	nonword	Pfanne	Tor	das	die	der
Mückenwerk*	**f-n**	nonword	Mücke	Werk	das	die	der
Burgschilf	**f-n**	nonword	Burg	Schilf	das	die	der
Walzenkalb*	**f-n**	nonword	Walze	Kalb	das	die	der
Treppengarn*	**f-n**	nonword	Treppe	Garn	das	die	der
Sockeninsekt*	**f-n**	nonword	Socke	Insekt	das	die	der
Urnensegel*	**f-n**	nonword	Urne	Segel	das	die	der
Stirnmosaik	**f-n**	nonword	Stirn	Mosaik	das	die	der
Glutschloss	**f-n**	nonword	Glut	Schloss	das	die	der
Lederschakal	**n-m**	nonword	Leder	Schakal	der	das	die
Stativquark	**n-m**	nonword	Stativ	Quark	der	das	die

Kameldom	**n-m**	nonword	Kamel	Dom	der	das	die
Messerelch	**n-m**	nonword	Messer	Elch	der	das	die
Cembalosaft	**n-m**	nonword	Cembalo	Saft	der	das	die
Tablettrock	**n-m**	nonword	Tablett	Rock	der	das	die
Wachsfrack	**n-m**	nonword	Wachs	Frack	der	das	die
Zebrastrand	**n-m**	nonword	Zebra	Strand	der	das	die
Glücksmund*	**n-m**	nonword	Glück	Mund	der	das	die
Pflastermond	**n-m**	nonword	Pflaster	Mond	der	das	die
Gummiluchs	**n-m**	nonword	Gummi	Luchs	der	das	die
Regalkran	**n-m**	nonword	Regal	Kran	der	das	die
Porträtofen	**n-m**	nonword	Porträt	Ofen	der	das	die
Steakkaktus	**n-m**	nonword	Steak	Kaktus	der	das	die
Datumsblitz*	**n-m**	nonword	Datum	Blitz	der	das	die
Kissenkrone	**n-f**	nonword	Kissen	Krone	die	das	der
Klaviersense	**n-f**	nonword	Klavier	Sense	die	das	der
Linealtrommel	**n-f**	nonword	Lineal	Trommel	die	das	der
Puzzlekette	**n-f**	nonword	Puzzle	Kette	die	das	der
Ticketangel	**n-f**	nonword	Ticket	Angel	die	das	der
Diadempizza	**n-f**	nonword	Diadem	Pizza	die	das	der
Beetschlange	**n-f**	nonword	Beet	Schlange	die	das	der
Liedertomate*	**n-f**	nonword	Lied	Tomate	die	das	der
Bleistaude	**n-f**	nonword	Blei	Staude	die	das	der
Wetterkreide	**n-f**	nonword	Wetter	Kreide	die	das	der
Rouletterübe	**n-f**	nonword	Roulette	Rübe	die	das	der
Badlupe	**n-f**	nonword	Bad	Lupe	die	das	der
Duoschorle	**n-f**	nonword	Duo	Schorle	die	das	der
Altersebbe*	**n-f**	nonword	Alter	Ebbe	die	das	der
Zimmerbrust	**n-f**	nonword	Zimmer	Brust	die	das	der
Besendelfin	**m-m**	nonword	Besen	Delfin	der	der	der
Fächerfrosch	**m-m**	nonword	Fächer	Frosch	der	der	der
Pilotendamm*	**m-m**	nonword	Pilot	Damm	der	der	der
Sesselaal	**m-m**	nonword	Sessel	Aal	der	der	der
Spatenefeu	**m-m**	nonword	Spaten	Efeu	der	der	der
Brunnenaltar	**m-m**	nonword	Brunnen	Altar	der	der	der
Buckelföhn	**m-m**	nonword	Buckel	Föhn	der	der	der
Sargfalke	**m-m**	nonword	Sarg	Falke	der	der	der
Traktorkopf	**m-m**	nonword	Traktor	Kopf	der	der	der
Sackdolch	**m-m**	nonword	Sack	Dolch	der	der	der
Radiobuffet	**n-n**	nonword	Radio	Buffet	das	das	das
Kreuzmädchen	**n-n**	nonword	Kreuz	Mädchen	das	das	das
Lamapult	**n-n**	nonword	Lama	Pult	das	das	das
Ponyraclette	**n-n**	nonword	Pony	Raclette	das	das	das
Skelettblau	**n-n**	nonword	Skelett	Blau	das	das	das
Brötchenheu	**n-n**	nonword	Brötchen	Heu	das	das	das
Zepterspiel	**n-n**	nonword	Zepter	Spiel	das	das	das
Caféjahr	**n-n**	nonword	Cafe	Jahr	das	das	das
Mineralwort	**n-n**	nonword	Mineral	Wort	das	das	das
Lebenshemd*	**n-n**	nonword	Leben	Hemd	das	das	das

Brückenhaut*	**f-f**	nonword	Brücke	Haut	die	die	die
Hexenkirsche*	**f-f**	nonword	Hexe	Kirsche	die	die	die
Pfeifeninsel*	**f-f**	nonword	Pfeife	Insel	die	die	die
Dosennonne*	**f-f**	nonword	Dose	Nonne	die	die	die
Möwenerbse*	**f-f**	nonword	Möwe	Erbse	die	die	die
Blusenamsel*	**f-f**	nonword	Bluse	Amsel	die	die	die
Oboenkrabbe*	**f-f**	nonword	Oboe	Krabbe	die	die	die
Milzbrosche	**f-f**	nonword	Milz	Brosche	die	die	die
Cremehupe	**f-f**	nonword	Creme	Hupe	die	die	die
Kiwicouch	**f-f**	nonword	Kiwi	Couch	die	die	die

Appendix B: English Translation of German Materials

Table B1: English translations of German compound word targets and their constituents.

Compound	Compound English	Modifier	Modifier English	Head	Head English
**Mülltonne**	garbage bin	Müll	garbage	Tonne	bin
**Schnabeltasse**	sippy cup	Schnabel	beak	Tasse	cup
**Windmühle**	windmill	Wind	wind	Mühle	mill
**Kaffeemaschine**	coffee machine	Kaffee	coffee	Maschine	machine
**Stockente**	mallard duck	Stock	stock	Ente	duck
**Nagelfeile**	nail file	Nagel	nail	Feile	file
**Käsetheke**	cheese counter	Käse	cheese	Theke	counter
**Sanduhr**	hourglass	Sand	sand	Uhr	clock
**Briefmarke**	stamp	Brief	letter	Marke	stamp
**Stachelbeere**	gooseberry	Stachel	sting	Beere	berry
**Latzhose**	dungarees	Latz	flap	Hose	pants
**Hundehütte***	kennel	Hund	dog	Hütte	hut
**Pudelmütze**	bobble hat	Pudel	poodle	Mütze	woolly hat
**Kreissäge**	circular saw	Kreis	circle	Säge	saw
**Rauchwolke**	cloud of smoke	Rauch	smoke	Wolke	cloud
**Eselsohr***	dog-ear	Esel	donkey	Ohr	ear
**Spiegelei**	fried egg	Spiegel	mirror	Ei	egg
**Schlauchboot**	dinghy	Schlauch	hose	Boot	boat
**Kleeblatt**	cloverleaf	Klee	clover	Blatt	leaf
**Armband**	wristband	Arm	arm	Band	band
**Zirkuszelt**	circus tent	Zirkus	circus	Zelt	tent
**Tischbein**	table leg	Tisch	table	Bein	leg
**Gürteltier**	armadillo	Gürtel	belt	Tier	animal
**Chorkonzert**	choir concert	Chor	choir	Konzert	concert
**Stromkabel**	electricity cable	Strom	electricity	Kabel	cable
**Teelicht**	tealight	Tee	tea	Licht	light
**Maishuhn**	corn-fed chicken	Mais	corn	Huhn	chicken
**Halstuch**	scarf	Hals	neck	Tuch	cloth
**Kürbiscurry**	pumpkin curry	Kürbis	pumpkin	Curry	curry
**Riesenrad***	ferris wheel	Riese	giant	Rad	wheel
**Rosenbusch***	rose	Rose	rose	Busch	bush
**Blumentopf***	flowerpot	Blume	flower	Topf	pot
**Geigenkasten***	violin case	Geige	violin	Kasten	case
**Lippenstift***	lip stick	Lippe	lip	Stift	pen
**Kassenbon***	receipt	Kasse	cash register	Bon	receipt
**Kirchturm***	steeple	Kirche	church	Turm	tower
**Federball**	badminton	Feder	feather	Ball	ball
**Sonnenschirm***	sun umbrella	Sonne	sun	Schirm	umbrella
**Handschuh**	glove	Hand	hand	Schuh	shoe
**Aktenschrank***	filing cabinet	Akte	file	Schrank	cabinet
**Kartoffelacker**	potato field	Kartoffel	potato	Acker	field
**Tannenbaum***	pine tree	Tanne	pine	Baum	tree
**Schulbus***	school bus	Schule	school	Bus	bus
**Bienenhonig***	bee honey	Biene	bee	Honig	honey

**Fliegenpilz***	fly agaric	Fliege	fly	Pilz	mushroom
**Butterbrot**	sandwich	Butter	butter	Brot	bread
**Tintenfass***	inkpot	Tinte	ink	Fass	pot
**Wespennest***	wasp nest	Wespe	wasp	Nest	nest
**Kunsthaar**	artificial hair	Kunst	art	Haar	hair
**Spinnennetz***	spider web	Spinne	spider	Netz	web
**Polizeiauto**	police car	Polizei	police	Auto	car
**Nudelsieb**	pasta strainer	Nudel	pasta	Sieb	strainer
**Prachtstück**	stunning piece	Pracht	glory	Stück	piece
**Wurzelgemüse**	root vegetable	Wurzel	root	Gemüse	vegetable
**Jagdgewehr**	hunting rifle	Jagd	hunt	Gewehr	rifle
**Schneckenhaus***	snail shell	Schnecke	snail	Haus	house
**Brautkleid**	wedding dress	Braut	bride	Kleid	dress
**Übungsheft***	exercise book	Übung	exercise	Heft	book
**Damenparfüm***	lady's parfum	Dame	lady	Parfüm	parfum
**Olivenöl***	olive oil	Olive	olive	Öl	oil
**Pferdeschwanz***	ponytail	Pferd	horse	Schwanz	tail
**Goldfisch**	goldfish	Gold	gold	Fisch	fish
**Geldschein**	banknote	Geld	money	Schein	note
**Eisbär**	ice bear	Eis	ice	Bär	bear
**Tennisprofi**	tennis pro	Tennis	tennis	Profi	pro
**Grashalm**	blade of grass	Gras	grass	Halm	straw
**Fleischwolf**	meat grinder	Fleisch	meat	Wolf	wolf
**Papierkorb**	paper bin	Papier	paper	Korb	basket
**Schiffsrumpf***	hull	Schiff	ship	Rumpf	hull
**Rohrzucker**	cane sugar	Rohr	cane	Zucker	sugar
**Benzinkanister**	petrol canister	Benzin	petrol	Kanister	canister
**Feldhase**	common hare	Feld	field	Hase	hare
**Glasteller**	glass plate	Glas	glass	Teller	plate
**Holzkamm**	wooden comb	Holz	wood	Kamm	comb
**Kinofilm**	movies	Kino	cinema	Film	movie
**Cellomusik**	cello music	Cello	cello	Musik	music
**Billiardkugel**	billiard ball	Billiard	billiard	Kugel	ball
**Gasmaske**	gas mask	Gas	gas	Maske	mask
**Büroklammer**	paper clip	Büro	office	Klammer	clip
**Silbermünze**	silver coin	Silber	silver	Münze	coin
**Telefonzelle**	telephone booth	Telefon	telephone	Zelle	booth
**Schildkröte**	turtle	Schild	shield	Kröte	toad
**Bierflasche**	beer bottle	Bier	beer	Flasche	bottle
**Landkarte**	map	Land	land	Karte	map
**Wasserwaage**	water scale	Wasser	water	Waage	scale
**Kanutour**	canoe tour	Kanu	canoe	Tour	tour
**Seilbahn**	cable car	Seil	rope	Bahn	train
**Wunderkerze**	sparkler	Wunder	miracle	Kerze	candle
**Augenklappe***	eye patch	Auge	eye	Klappe	patch
**Klobrille**	toilet seat	Klo	toilet	Brille	glasses
